# (*E*)-4-Chloro-*N*-[4-(methyl­sulfon­yl)benzyl­idene]aniline

**DOI:** 10.1107/S160053681105361X

**Published:** 2011-12-17

**Authors:** Yue-Hu Chen, Fang Wang, Guo-Qiang Li, Shao-Song Qian

**Affiliations:** a School of Life Sciences, ShanDong University of Technology, ZiBo 255049, People’s Republic of China

## Abstract

In the crystal structure of the title compound, C_14_H_12_ClNO_2_S, the mol­ecules display a *trans* conformation with respect to the C=N double bond. The dihedral angle between the methyl­sulfonyl benzene and chloro­benzene rings is 59.59 (8)°. The crystal packing is stabilized by weak C—H⋯O inter­actions and by π–π stacking inter­actions between inversion-related methyl­sulfonyl benzene rings [centroid–centroid distance = 3.8579 (11) Å].

## Related literature

For background to the pharmacological properties of Schiff base compounds, see: Villar *et al.* (2004 ▶); Pandey *et al.* (1999 ▶); Singh & Dash (1988 ▶). For related structures, see: Qian & Cui (2009 ▶); Qian & Liu (2010 ▶).
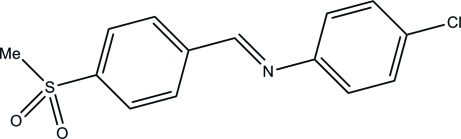


## Experimental

### 

#### Crystal data

C_14_H_12_ClNO_2_S
*M* *_r_* = 293.77
Monoclinic, 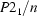

*a* = 8.6206 (10) Å
*b* = 8.8748 (10) Å
*c* = 17.799 (2) Å
β = 94.972 (1)°
*V* = 1356.6 (3) Å^3^
*Z* = 4
Mo *K*α radiation
μ = 0.43 mm^−1^
*T* = 296 K
0.25 × 0.23 × 0.21 mm

#### Data collection

Bruker APEXII CCD diffractometer
Absorption correction: multi-scan (*SADABS*; Bruker, 2004 ▶) *T*_min_ = 0.898, *T*_max_ = 0.913
11492 measured reflections
3320 independent reflections
2596 reflections with *I* > 2σ(*I*)
*R*_int_ = 0.040

#### Refinement

*R*[*F* ^2^ > 2σ(*F* ^2^)] = 0.038
*wR*(*F* ^2^) = 0.110
*S* = 1.04
3320 reflections
173 parameters
H-atom parameters constrained
Δρ_max_ = 0.29 e Å^−3^
Δρ_min_ = −0.40 e Å^−3^

### 

Data collection: *APEX2* (Bruker, 2004 ▶); cell refinement: *SAINT* (Bruker, 2004 ▶); data reduction: *SAINT*; program(s) used to solve structure: *SHELXS97* (Sheldrick, 2008 ▶); program(s) used to refine structure: *SHELXL97* (Sheldrick, 2008 ▶); molecular graphics: *XP* in *SHELXTL* (Sheldrick, 2008 ▶); software used to prepare material for publication: *SHELXTL*.

## Supplementary Material

Crystal structure: contains datablock(s) global, I. DOI: 10.1107/S160053681105361X/pk2372sup1.cif

Structure factors: contains datablock(s) I. DOI: 10.1107/S160053681105361X/pk2372Isup2.hkl

Supplementary material file. DOI: 10.1107/S160053681105361X/pk2372Isup3.cml

Additional supplementary materials:  crystallographic information; 3D view; checkCIF report

## Figures and Tables

**Table 1:** Hydrogen-bond geometry (Å, °)

*D*—H⋯*A*	*D*—H	H⋯*A*	*D*⋯*A*	*D*—H⋯*A*
C7—H7⋯O1^i^	0.93	2.56	3.126 (2)	120

Symmetry code: (i) 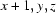.

**Fig. 1.**
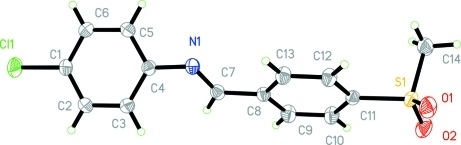
The molecular structure of the title compound. Displacement ellipsoids are drawn at the 30% probability level.

**Fig. 2.**
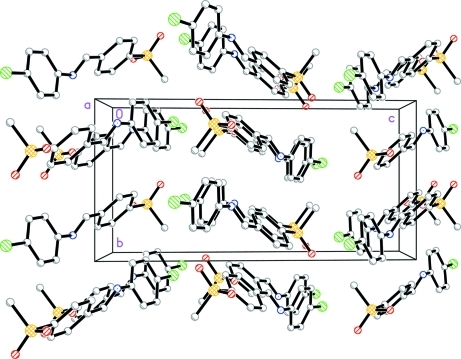
A packing diagram for the title compound, viewed down the a axis.

## supplementary crystallographic information

### Comment

Schiff base compounds have been of great interest for many years due to their wide range of biological activities. They have been reported to possess pharmacological activity, including anticancer (Villar *et al.*, 2004), antibacterial (Pandey *et al.*, 1999), and antifungal (Singh & Dash, 1988) properties.  As an extension of our work on structural characterization of Schiff base compounds, we report here the crystal structure of the title compound (Fig. 1).  All bond lengths are comparable to the values observed in closely related compounds (Qian & Cui, 2009; Qian & Liu, 2010).  The title compound displays a trans-configuration with respect to the C=N double bond.  The dihedral angle between the methylsulfonyl benzene and chlorobenzene rings is 59.59 (8)°.  The crystal packing (Fig. 2) is stabilized by weak C—H···O interactions and by π-π stacking interactions of inversion-related (1-x, 1-y, 2-z) methylsulfonyl benzene rings [centroid-centroid distance = 3.8579 (11)Å].

### Experimental

4-(Methylsulfonyl)benzaldehyde (0.184 g) and 4-chloroaniline (0.114 g) were dissolved in acetonitrile (20 ml). The mixture was stirred at room temperature for 15 min to give a clear yellow solution. After sitting for 5 days exposed to air, yellow block-shaped crystals were obtained at the bottom of the vessel.

### Refinement

All H atoms were placed in geometrical positions and constrained to ride on their parent atoms with C—H distances in the range 0.93–0.96 Å. They were treated as riding atoms, with *U*_iso_(H) = k*U*_eq_(C), where k = 1.5 for methyl and 1.2 for all other H atoms.

### Crystal data

**Table:**

C_14_H_12_ClNO_2_S	*F*(000) = 608
*M**_r_* = 293.77	*D*_x_ = 1.438 Mg m^−^^3^
Monoclinic, *P*2_1_/*n*	Mo *K*α radiation, λ = 0.71073 Å
Hall symbol: -P 2yn	Cell parameters from 3320 reflections
*a* = 8.6206 (10) Å	θ = 2.3–28.3°
*b* = 8.8748 (10) Å	µ = 0.43 mm^−^^1^
*c* = 17.799 (2) Å	*T* = 296 K
β = 94.972 (1)°	Block, yellow
*V* = 1356.6 (3) Å^3^	0.25 × 0.23 × 0.21 mm
*Z* = 4	

### Data collection

**Table:**

Bruker APEXII CCD diffractometer	3320 independent reflections
Radiation source: fine-focus sealed tube	2596 reflections with *I* > 2σ(*I*)
graphite	*R*_int_ = 0.040
φ and ω scans	θ_max_ = 28.3°, θ_min_ = 2.3°
Absorption correction: multi-scan (*SADABS*; Bruker, 2004)	*h* = −11→9
*T*_min_ = 0.898, *T*_max_ = 0.913	*k* = −11→11
11492 measured reflections	*l* = −23→23

### Refinement

**Table:**

Refinement on *F*^2^	Primary atom site location: structure-invariant direct methods
Least-squares matrix: full	Secondary atom site location: difference Fourier map
*R*[*F*^2^ > 2σ(*F*^2^)] = 0.038	Hydrogen site location: inferred from neighbouring sites
*wR*(*F*^2^) = 0.110	H-atom parameters constrained
*S* = 1.04	*w* = 1/[σ^2^(*F*_o_^2^) + (0.0507*P*)^2^ + 0.4568*P*] where *P* = (*F*_o_^2^ + 2*F*_c_^2^)/3
3320 reflections	(Δ/σ)_max_ = 0.002
173 parameters	Δρ_max_ = 0.29 e Å^−^^3^
0 restraints	Δρ_min_ = −0.40 e Å^−^^3^

### Special details

**Table:**

Geometry. All esds (except the esd in the dihedral angle between two l.s. planes) are estimated using the full covariance matrix. The cell esds are taken into account individually in the estimation of esds in distances, angles and torsion angles; correlations between esds in cell parameters are only used when they are defined by crystal symmetry. An approximate (isotropic) treatment of cell esds is used for estimating esds involving l.s. planes.
Refinement. Refinement of F^2^ against ALL reflections. The weighted R-factor wR and goodness of fit S are based on F^2^, conventional R-factors R are based on F, with F set to zero for negative F^2^. The threshold expression of F^2^ > 2sigma(F^2^) is used only for calculating R-factors(gt) etc. and is not relevant to the choice of reflections for refinement. R-factors based on F^2^ are statistically about twice as large as those based on F, and R- factors based on ALL data will be even larger.

### Fractional atomic coordinates and isotropic or equivalent isotropic displacement parameters (Å^2^)

**Table:**

	*x*	*y*	*z*	*U*_iso_*/*U*_eq_	
Cl1	1.44038 (7)	0.87940 (7)	0.77377 (3)	0.06397 (19)	
S1	0.25645 (5)	0.66162 (5)	1.12920 (2)	0.03972 (14)	
O1	0.12146 (16)	0.67285 (18)	1.07669 (8)	0.0580 (4)	
O2	0.27398 (19)	0.52896 (17)	1.17454 (9)	0.0644 (4)	
N1	0.85437 (18)	0.84443 (17)	0.92329 (9)	0.0414 (3)	
C1	1.2692 (2)	0.8676 (2)	0.81840 (10)	0.0403 (4)	
C2	1.1903 (2)	0.7325 (2)	0.81801 (10)	0.0431 (4)	
H2	1.2286	0.6487	0.7943	0.052*	
C3	1.0540 (2)	0.7226 (2)	0.85314 (10)	0.0407 (4)	
H3	1.0004	0.6316	0.8532	0.049*	
C4	0.9963 (2)	0.84819 (19)	0.88855 (9)	0.0370 (4)	
C5	1.0771 (2)	0.9831 (2)	0.88774 (10)	0.0431 (4)	
H5	1.0384	1.0679	0.9105	0.052*	
C6	1.2147 (2)	0.9930 (2)	0.85339 (11)	0.0447 (4)	
H6	1.2697	1.0832	0.8539	0.054*	
C7	0.8294 (2)	0.7307 (2)	0.96360 (9)	0.0386 (4)	
H7	0.9046	0.6554	0.9693	0.046*	
C8	0.68621 (19)	0.71368 (19)	1.00167 (9)	0.0358 (4)	
C9	0.6839 (2)	0.6093 (2)	1.05955 (11)	0.0475 (4)	
H9	0.7711	0.5498	1.0722	0.057*	
C10	0.5532 (2)	0.5927 (2)	1.09869 (11)	0.0465 (4)	
H10	0.5526	0.5232	1.1378	0.056*	
C11	0.42363 (19)	0.68041 (18)	1.07919 (9)	0.0350 (3)	
C12	0.4227 (2)	0.7835 (2)	1.02035 (10)	0.0434 (4)	
H12	0.3342	0.8404	1.0067	0.052*	
C13	0.5543 (2)	0.8005 (2)	0.98253 (10)	0.0431 (4)	
H13	0.5551	0.8708	0.9438	0.052*	
C14	0.2630 (3)	0.8207 (2)	1.18768 (12)	0.0544 (5)	
H14A	0.1760	0.8191	1.2179	0.082*	
H14B	0.2586	0.9102	1.1572	0.082*	
H14C	0.3582	0.8201	1.2200	0.082*	

### Atomic displacement parameters (Å^2^)

**Table:**

	*U*^11^	*U*^22^	*U*^33^	*U*^12^	*U*^13^	*U*^23^
Cl1	0.0498 (3)	0.0688 (4)	0.0779 (4)	−0.0003 (2)	0.0313 (3)	0.0035 (3)
S1	0.0346 (2)	0.0412 (2)	0.0442 (2)	−0.00697 (17)	0.00761 (17)	−0.00031 (17)
O1	0.0350 (7)	0.0783 (10)	0.0600 (8)	−0.0089 (7)	0.0000 (6)	−0.0115 (7)
O2	0.0652 (10)	0.0534 (9)	0.0780 (10)	−0.0046 (7)	0.0263 (8)	0.0217 (8)
N1	0.0337 (8)	0.0431 (8)	0.0484 (8)	−0.0006 (6)	0.0101 (6)	−0.0019 (6)
C1	0.0345 (9)	0.0471 (10)	0.0403 (9)	0.0007 (7)	0.0079 (7)	0.0022 (7)
C2	0.0439 (10)	0.0415 (9)	0.0452 (9)	0.0039 (8)	0.0104 (8)	−0.0045 (8)
C3	0.0399 (10)	0.0380 (9)	0.0447 (9)	−0.0031 (7)	0.0065 (7)	−0.0027 (7)
C4	0.0315 (9)	0.0407 (9)	0.0389 (8)	0.0007 (7)	0.0037 (7)	−0.0004 (7)
C5	0.0433 (10)	0.0391 (9)	0.0479 (10)	0.0000 (7)	0.0102 (8)	−0.0064 (7)
C6	0.0448 (10)	0.0403 (9)	0.0498 (10)	−0.0082 (8)	0.0093 (8)	−0.0007 (8)
C7	0.0324 (9)	0.0427 (9)	0.0406 (8)	0.0014 (7)	0.0033 (7)	−0.0034 (7)
C8	0.0331 (9)	0.0368 (8)	0.0377 (8)	−0.0001 (7)	0.0046 (7)	−0.0026 (7)
C9	0.0393 (10)	0.0498 (11)	0.0540 (11)	0.0126 (8)	0.0065 (8)	0.0127 (8)
C10	0.0436 (10)	0.0471 (10)	0.0496 (10)	0.0074 (8)	0.0090 (8)	0.0161 (8)
C11	0.0323 (8)	0.0356 (8)	0.0371 (8)	−0.0016 (6)	0.0037 (6)	0.0003 (6)
C12	0.0333 (9)	0.0470 (10)	0.0500 (10)	0.0062 (7)	0.0047 (7)	0.0123 (8)
C13	0.0387 (9)	0.0458 (10)	0.0451 (9)	0.0040 (7)	0.0060 (7)	0.0130 (8)
C14	0.0500 (12)	0.0614 (13)	0.0531 (11)	−0.0075 (9)	0.0120 (9)	−0.0155 (10)

### Geometric parameters (Å, °)

**Table:**

Cl1—C1	1.7389 (18)	C6—H6	0.9300
S1—O2	1.4281 (15)	C7—C8	1.467 (2)
S1—O1	1.4315 (15)	C7—H7	0.9300
S1—C14	1.752 (2)	C8—C9	1.387 (2)
S1—C11	1.7662 (17)	C8—C13	1.391 (2)
N1—C7	1.267 (2)	C9—C10	1.383 (3)
N1—C4	1.418 (2)	C9—H9	0.9300
C1—C2	1.377 (3)	C10—C11	1.381 (3)
C1—C6	1.378 (3)	C10—H10	0.9300
C2—C3	1.382 (2)	C11—C12	1.390 (2)
C2—H2	0.9300	C12—C13	1.377 (2)
C3—C4	1.393 (2)	C12—H12	0.9300
C3—H3	0.9300	C13—H13	0.9300
C4—C5	1.386 (2)	C14—H14A	0.9600
C5—C6	1.384 (3)	C14—H14B	0.9600
C5—H5	0.9300	C14—H14C	0.9600
			
O2—S1—O1	117.81 (10)	N1—C7—H7	118.8
O2—S1—C14	109.41 (11)	C8—C7—H7	118.8
O1—S1—C14	108.32 (10)	C9—C8—C13	119.20 (16)
O2—S1—C11	108.16 (9)	C9—C8—C7	118.61 (15)
O1—S1—C11	108.49 (8)	C13—C8—C7	122.18 (15)
C14—S1—C11	103.73 (9)	C10—C9—C8	120.66 (17)
C7—N1—C4	117.42 (15)	C10—C9—H9	119.7
C2—C1—C6	121.20 (17)	C8—C9—H9	119.7
C2—C1—Cl1	119.21 (14)	C11—C10—C9	119.35 (16)
C6—C1—Cl1	119.59 (14)	C11—C10—H10	120.3
C1—C2—C3	119.48 (16)	C9—C10—H10	120.3
C1—C2—H2	120.3	C10—C11—C12	120.79 (16)
C3—C2—H2	120.3	C10—C11—S1	119.85 (13)
C2—C3—C4	120.29 (16)	C12—C11—S1	119.36 (13)
C2—C3—H3	119.9	C13—C12—C11	119.31 (16)
C4—C3—H3	119.9	C13—C12—H12	120.3
C5—C4—C3	119.17 (16)	C11—C12—H12	120.3
C5—C4—N1	118.56 (15)	C12—C13—C8	120.67 (16)
C3—C4—N1	122.23 (15)	C12—C13—H13	119.7
C6—C5—C4	120.69 (17)	C8—C13—H13	119.7
C6—C5—H5	119.7	S1—C14—H14A	109.5
C4—C5—H5	119.7	S1—C14—H14B	109.5
C1—C6—C5	119.14 (17)	H14A—C14—H14B	109.5
C1—C6—H6	120.4	S1—C14—H14C	109.5
C5—C6—H6	120.4	H14A—C14—H14C	109.5
N1—C7—C8	122.31 (16)	H14B—C14—H14C	109.5

### Hydrogen-bond geometry (Å, °)

**Table:**

*D*—H···*A*	*D*—H	H···*A*	*D*···*A*	*D*—H···*A*
C7—H7···O1^i^	0.93	2.56	3.126 (2)	120.

Symmetry codes: (i) *x*+1, *y*, *z*.
